# Matrine Is Identified as a Novel Macropinocytosis Inducer by a Network Target Approach

**DOI:** 10.3389/fphar.2018.00010

**Published:** 2018-01-26

**Authors:** Bo Zhang, Xin Wang, Yan Li, Min Wu, Shu-Yan Wang, Shao Li

**Affiliations:** ^1^MOE Key Laboratory of Bioinformatics, TCM-X Center, Bioinformatics Division, TNLIST, Department of Automation, Tsinghua University, Beijing, China; ^2^Tianjin International Joint Academy of Biotechnology and Medicine, Tianjin, China; ^3^Institute of Materia Medica, Chinese Academy of Medical Sciences, Peking Union Medical College, Beijing, China

**Keywords:** matrine, macropinocytosis, network target, network pharmacology, natural products

## Abstract

Comprehensively understanding pharmacological functions of natural products is a key issue to be addressed for the discovery of new drugs. Unlike some single-target drugs, natural products always exert diverse therapeutic effects through acting on a “network” that consists of multiple targets, making it necessary to develop a systematic approach, e.g., network pharmacology, to reveal pharmacological functions of natural products and infer their mechanisms of action. In this work, to identify the “network target” of a natural product, we perform a functional analysis of matrine, a marketed drug in China extracted from a medical herb *Ku-Shen* (*Radix Sophorae Flavescentis*). Here, the network target of matrine was firstly predicted by drugCIPHER, a genome-wide target prediction method. Based on the network target of matrine, we performed a functional gene set enrichment analysis to computationally identify the potential pharmacological functions of matrine, most of which are supported by the literature evidence, including neurotoxicity and neuropharmacological activities of matrine. Furthermore, computational results demonstrated that matrine has the potential for the induction of macropinocytosis and the regulation of ATP metabolism. Our experimental data revealed that the large vesicles induced by matrine are consistent with the typical characteristics of macropinosome. Our verification results also suggested that matrine could decrease cellular ATP level. These findings demonstrated the availability and effectiveness of the network target strategy for identifying the comprehensive pharmacological functions of natural products.

## Introduction

Structurally diverse compounds from natural products have historically been used as therapeutic agents for disease control and prevention and as a fertile source of lead compounds for the development of novel drugs (Cordell, 2014). However, a more thorough use of natural products for drug discovery is hampered by a lack of a complete mechanistic understanding of their pharmacological functions. With the rising of natural products as drug candidates, comprehensively determining small molecule compound–target interaction profiles have become increasingly necessary. Target and mechanism study of natural products is a central requirement for understanding the complexity of natural products and developing new drugs (Xu, 2011). Traditionally, identification strategies of targets have included genomic, biochemical and chemical biology-based approaches (Farha and Brown, 2016). However, more alternative theoretical and practical approaches are still required to better honed for natural product discovery.

To comprehensively understand the pharmacological functions of natural products, we for the first time proposed a new therapeutic concept “network target,” a core idea that is distinct from the current “one target, one drug” paradigm often associated with reductionist medicine (Li, 2011; Li et al., 2011). From the perspective of the network target, natural products can target a biological network of several key molecules that derived from the multi-target nature of traditional medicine (Li et al., 2007; Li, 2009). The network target concept represents a description of complex mechanism of drug action, which suggests that the action of many small molecule compounds may have diverse effects on many proteins, leading to a perturbation of biological networks at different levels (including changes in gene expression, post-translational modifications, protein–protein interactions and metabolites) (Li and Zhang, 2013). Furthermore, the enriched biological processes associated with a set of interacting targets in a perturbed network target can be used to unveil the pleiotropic effects of natural products (Li et al., 2014; Liang et al., 2014). At the same time, the network pharmacology is evolving as a holistic strategy for drug research and development by integrating information science and systematic medicine (Hopkins, 2007; Li, 2015). Recent advances in experimental and computational strategies, such as chemogenomics and *in silico* approach, have provided big biological data of natural products toward network pharmacology, yielding important insights into pharmacological actions (Brehme et al., 2009; Zhao and Iyengar, 2012; Qi et al., 2016) and toxicological effects (Zhang et al., 2015). Accordingly, the network-target-based network pharmacology for a natural product is now critical to provide precise and complete functional characterization that could be useful for drug discovery and development.

Recently, a proven group of Chinese patent medicines has held and still holds an important position in the treatment of a variety of cancers in China, and statistical analysis results from nationwide medical insurance data have suggested that *Sophorae Flavescentis Radix*, namely *Ku-Shen*, is widely used in the treatment of a variety of cancers in China (Wu et al., 2015). Matrine is a main active constituent in *Ku-Shen* which has been approved as herbal medicine in China, and has polypharmacology including anti-inflammatory (Zhang B. et al., 2011; Wu G. et al., 2017), anti-tumor (Wu J. et al., 2017), anti-angiogenic (Li et al., 2010) and so on. It is a kind of quinolizidine alkaloid compound and very similar in structure to oxymatrine and sophoridine. Yet, despite considerable previous research and development efforts to date, functional characterization of matrine endures a wonderful and complex challenge for pharmacologists.

Trying to approach the comprehensive mechanisms of matrine, here we took a systematic approach based on targets prediction and network target analysis to identifying the network target and functional characterization of matrine. Taking advantage of the extensive literature and experimental evidence, we systematically examined the effects of matrine on different biological processes involved and identified the network target of matrine. The influence of network target by matrine offers a unified molecular mechanism for the diverse pharmacological actions of matrine. Our preliminary evidence also revealed a previously unknown activity of matrine, induction of macropinocytosis, which has important implication in the therapeutic efficacy of *Ku-Shen*.

## Materials and Methods

### Computational Procedure

#### Target Prediction, Network Target and Functional Enrichment Analysis for Matrine

The potential targets were predicted by the drugCIPHER-CS method (Zhao and Li, 2010). The drugCIPHER-CS method uses a regression model to predict relationships between compounds and proteins by correlating the closeness of the global pharmacological network and the protein–protein interaction network. DrugCIPHER-CS calculated the correlation between the chemical similarity vector of matrine-seed drugs and drug-protein closeness vector as the likelihood of the interactions of drug-target. Accordingly, we define the similarity vector **CS**_d_ for matrine *d* as {*CS*_dd1_, *CS*_dd2,_ …, *CS*_ddn_} and extend equation (1) into:

(1) Φp= β′p+∑dj∈ B(p) α′CpdjSdj

where *d*_j_ is the known drug *j* binding to the given protein *p*. β′_p_ and α′_pdj_ are the model coefficients. The likelihood between matrine *d* and protein *p* is defined as concordance score:

(2) $$\rho_{pd}=\frac{cov(CS_{d},\Phi_{p})}{\sigma (CS_{d})\sigma (\Phi_{p})}\;\left(2\right)$$

According to the concordance score of each target protein for matrine, drugCIPHER-CS prioritizes the proteins in the PPI network, and the candidate proteins with high concordance score are hypothesized to be predicted targets of matrine with high confidence.

As the top 100 predicted targets of drug prediction can achieve the high prediction accuracy (77.3%), the network target of matrine is composed of the top 100 predicted targets of matrine in the protein–protein interaction network (Zhao and Li, 2010). To gain insight into the detailed functions of the matrine, we examined the overrepresented biological processes of the network target of matrine using the Database for Annotation, Visualization and Integrated Discovery (DAVID) (Huang et al., 2009). The network target of matrine was mainly examined for enrichment Gene Ontology (GO) biological process (BP) (Supplementary Table S1) (Ashburner et al., 2000). Here, we reserved all the enriched biological processes. The steps for constructing BP network were depicted as followed. First, PPI network was generated with 137,037 interactions among 13,388 (Zhao and Li, 2010). Then, we used gene sets of enriched biological processes of matrine to generate a sub network. Finally, an edge will be added between two classes of biological processes if there are shared genes in two classes.

### Experimental Validation

#### Reagents and Cell Culture

Matrine and amiloride hydrochloride was purchased from Sigma (Shanghai, China). Lucifer yellow (LY), pHrodo^TM^ Green Dextran and Alexa Fluor^®^488 Phalloidin were purchased from Invitrogen (Shanghai, China). EIPA was purchased from Tocris. HepG2, PLC, HCT-8, HCT-116, HT29 and DLD-1 were obtained from ATCC (American Type Culture Collection, Rockville, MD, United States).

#### Transmission Electron Microscopy

Human colon cancer DLD-1 cells were exposed to 1.25, 2.5 and 5 mM matrine for 12 h and then fixed, dehydrated, and infiltrated for Transmission Electron Microscopy (TEM) as described previously (Kitambi et al., 2014). Ultrathin sections were collected on copper 300-mesh support grids, stained with uranyl acetate and lead citrate, and examined under a Hitachi H-7650B TEM.

#### Time-Lapse Microscopy

DLD-1 cells (200,000 cells) were plated in a 35-mm glass-bottom microwell culture dish. One day after plating, cells were treated with 5 mM matrine in RPMI-1640 medium with 10% FBS. The dish was immediately placed in a humidified Live Cell chamber (Pathology Devices, Westminster, MD, United States) equilibrated with 5% CO_2_ at 37°C. The chamber was placed on the stage of a Nikon Eclipse Ti inverted microscope, equipped with a digital camera and Slide-book software (Intelligent Imaging Innovations, Inc., Denver, CO, United States). The software was set to automatically acquire phase-contrast images every 1 s for the indicated period of time.

#### The Uptake of Fluid-Phase Fluorescent Tracers

Labeling of endocytic compartments with these fluid-phase tracers was performed as previously described (Overmeyer et al., 2008). Briefly, cells were incubated with LY (500 μg/ml in the phenol red-free DMEM containing 10% fetal bovine serum) and pHrodo^TM^ Green dextran (50 μg/ml in the same medium) for 5 h and 10 h in a 37°C, 5% CO_2_ incubator, respectively. The tracer was removed and the cells were washed once or twice with the medium. Phase-contrast and fluorescent images of the living cells were acquired on a Nikon Eclipse Ti fluorescence microscope with a digital camera and NIS-Elements AR software (Nikon Instruments, Inc., Melville, NY, United States).

#### Confocal Fluorescence Microscopy

For colocalization experiments, DLD-1 cells that had been treated with matrine were prepared for immunofluorescence microscopy as described previously (Kitambi et al., 2014). Phalloidin-FITC to detect cytoskeletons was purchased from Sigma. The cells were examined by Nikon A1+/A1R+ confocal microscopy.

#### Treatment of Cells with EIPA and Amiloride

To inhibit macropinocytosis, DLD-1 cells were washed twice with PBS and then pretreated for 30 min with RPMI-1640 + 0.5% BSA in the presence or absence of 50 μM EIPA or 4 mM Amiloride. Following pretreatment, matrine was added to the dishes at a final concentration of 5 mM and phase-contrast images were acquired 5 h later.

#### Statistical Analysis

At least three independent experiments were conducted in each study. The values are expressed as the means ± SEM. A two-tailed *t*-test was performed to determine the statistical significance. *P* < 0.05 was considered significant.

## Results

### The Network Target and Literature Validation of Matrine

**FIGURE 1:**
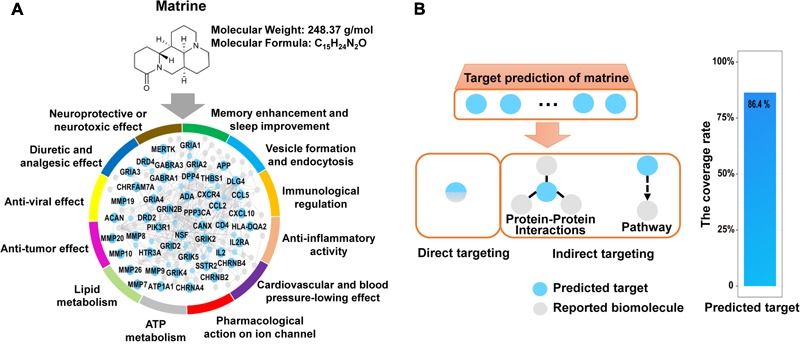
Target prediction results of matrine and extensive literature verification. **(A)** Matrine-targeting molecular network with annotation labels comprising GO BP classes. **(B)** The coverage rate of the predicted targets of matrine toward reported biomolecules, which was calculated by [the intersection of the predicted targets and reported biomolecules/the number of reported biomolecules] × 100%. There are two main relationships between the predicted targets and reported biomolecules, direct and indirect association. Direct association represents that the predicted targets are identical with reported biomolecules. Indirect association represents that the predicted targets are linked to reported biomolecules by protein–protein interactions or pathways.

Actually, matrine is one of several alkaloids in *Ku-Shen* that has not only been experimentally shown to cause a neurotoxic response, but has been shown to exert neuropharmacological activities, e.g., antinociceptive effects, hypothermic actions, neuroprotective role, antiepileptic effects, sedative effects, memory enhancement and sleep improvement. In addition, matrine may serve other pleiotropic functions, such as immunological regulation, anti-inflammatory activity, cardiac effects and anti-tumor effect (Supplementary Table S1). Therefore, we hope to construct a simple, rapid and lower-cost strategy on overall interpretation of biological functions of natural products like matrine. Here, the network target of matrine was performed by our network-based method drugCIPHER (Zhao and Li, 2010). We selected top 100 predicted targets in the protein–protein interaction network as the network target of matrine. Further, we examined the overrepresented ontological terms of the network target using enrichment analysis (Huang et al., 2009). Expectedly, the most representative GO BP terms of the network target of matrine include “neurological system process,” “sensory perception of pain,” “learning or memory,” “immune response,” “inflammatory response,” “regulation of heart contraction,” “tissue remodeling,” “ion homeostasis,” “regulation of lipase activity,” “regulation of blood pressure,” “response to oxidative stress,” “regulation of cell proliferation,” “regulation of apoptosis,” “cell migration,” “angiogenesis,” “leukocyte differentiation,” and “viral reproduction,” thus validating the reliability of the predicted targets given the well-documented pharmacological and biological effects of matrine (**Figure 1A**).

It is true that bioactive natural products can essentially regulate a molecular network by binding multiple targets, which may imply possible multiple effects. Therefore, to obtain a comprehensive functional characterization of matrine, we firstly validated the reliability of the predicted molecular network regulated by matrine. Here, we searched for reported molecular mechanisms related to matrine from Pubmed and CNKI database by the way of literature mining. The predicted targets of matrine can be connected to its reported biomolecules by direct or indirect targeting (protein–protein interactions or signaling pathways) (Rubio-Perez et al., 2015). The results suggested that the predicted targets of matrine can be related to the molecular mechanisms reported in the literature. The statistical results showed that the predicted targets of matrine can cover 86.4% of the reported molecular mechanisms and the reliability of the predicted targets of matrine was found to be very high, which can be used for the comprehensive functional characterization of matrine (**Figure 1B**).

### Comprehensive Functional Assignment and Preliminary Validation of Matrine

It has been reported that matrine possesses a variety of pharmacological effects. Besides, oral administration of large doses of *Ku-Shen* is reported to cause toxicity and side effects, including salivation, tachycardia and abnormal gait (Drew et al., 2002). Larger doses that induce more severe poisoning may result in central nervous system (CNS) excitement with muscle spasm and seizures, followed by signs of CNS (primarily respiratory) depression with a decreased respiratory rate and promotes oligodendrocyte development, potentially progressing to apnea (Kamei et al., 1997; Liu et al., 2017). Therefore, we need to provide an analysis strategy based on the network target of matrine that derive a set of biological functions for revealing the bioactive diversity and side effects of matrine.

**FIGURE 2:**
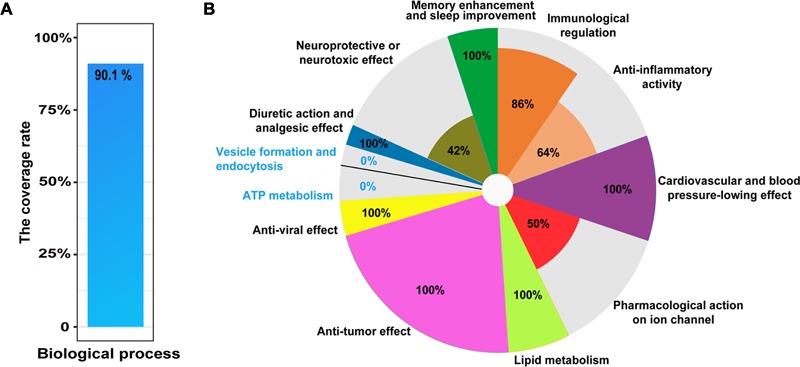
Literature verification of the GO terms in BP category at all levels enriched from the network target of matrine. **(A)** The coverage rate of potential biological processes with literature evidence for matrine, which is defined as [the intersection of reported GO terms and enriched GO terms/the number of reported GO terms] × 100%. **(B)** Pie chart for the coverage rate of enriched GO terms in different classes from predicted targets of matrine covered by known biological processes of matrine. The coverage rate of each sector is calculated by [the intersection of reported GO terms and enriched GO terms/the number of enriched GO terms in one class] × 100%.

To explore comprehensively functional annotations of the matrine, all of enriched terms, regardless of *P*-value, are shown in Supplementary Table S1. Enrichment analysis results showed that the network target of matrine are involved in neurological system processes, which may substantially disturb these biological processes. Indeed, the top 10 GO BP terms in the functional enrichment list significantly focused on nervous system, for example, synaptic transmission (*P* = 1.41E-15) as well as the transmission of nerve impulses (*P* = 2.92E-15). The results of literature validation suggested that many enriched biological processes have been reported in the literature (**Table 1**). Further, to evaluate this enrichment results, we manually collected 101 literatures related to biological processes of matrine. The results showed that 90.1% of reported biological processes were covered by enriched biological processes of the network target of matrine (**Figure 2A**). The representative biological functions of matrine are summarized in **Table 1** and **Figure 2B**. Anti-tumor effect, anti-viral effect, lipid metabolism, cardiovascular and blood pressure-lowing effect in the enriched biological functions of predicted targets of matrine are completely supported by literature evidence. Other important enriched biological functions of matrine can also be partly validated. These results suggested that featured as high-throughput, computational and capable of rapid analyses, our network pharmacology approach would have a strong potential to be applicable to other natural products for precise and comprehensive functional characterization.

**Table 1:** Enriched biological processes of predicted targets for matrine.

Biological process class	Gene ontology term	Reference
Neuroprotective or neurotoxic effect	Synaptic transmission	Yin and Zhu, 2005
	Neurological system process	Lu et al., 2014
	Glutamate signaling pathway	Zhang and Song, 2014
Analgesic effect	Sensory perception of pain	Wang et al., 2013
Memory enhancement and sleep improvement	Learning or memory	Cui et al., 2017
	Rhythmic process	Liu and Li, 2012
Lipid and energy metabolism	Fatty acid metabolic process	Zeng et al., 2015
	ATP metabolic process	NA
Pharmacological action on ion channel	Ion transport	Wei et al., 2013
Anti-inflammatory and immunological regulation	Inflammatory response	Zhang B. et al., 2011
	Lymphocyte activation	Zhao et al., 2011
Cardiovascular effect	Regulation of catecholamine secretion	Wang and Liu, 2012
	Tissue remodeling	Zhang et al., 2006
Anti-viral effect	Viral reproduction	Li et al., 2005
Vesicle formation and endocytosis	Membrane invagination	NA
	Endocytosis	NA
Anti-tumor effect	Regulation of cell proliferation	Liang et al., 2012
	Biological adhesion	Zhang et al., 2013
	Angiogenesis	Liu et al., 2014
	Cell migration	Wang et al., 2017

**FIGURE 3:**
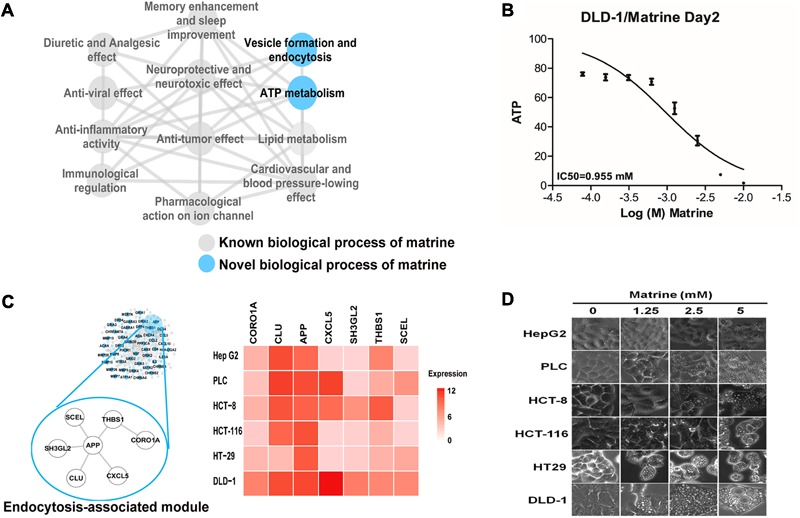
Identification and preliminary validation of novel functions of matrine. **(A)** Biological process network represents the comprehensive functional characterization of matrine using our network pharmacology approach. Blue nodes represent unreported biological processes of matrine. **(B)** Dose-effect relationship of matrine on cellular ATP level. **(C)** The endocytosis-associated network module of matrine with expression in different cancer cell lines. **(D)** Cell phenotype changing after different cancer cells treated with matrine (1.25–5 μM).

Multiple and complex actions of natural products can be described in terms of molecular networks capturing the intricate web of connections among their targets (Burkard et al., 2010). To further explore the functional characterization of matrine on a large scale, we constructed an enriched functional network for better interpreting the global functional connections of matrine (**Figure 3A**). The structure of this functional network shown that known and novel GO BP functions share the same molecular mechanisms. Despite being a drug with well-characterized pharmacological actions, matrine has never been previously linked to ATP metabolism and membrane organization/endocytosis. To verify the effect of matrine on the ATP metabolism, we evaluated the ATP levels in the DLD-1 cells treated with matrine by a well-established assay for the levels of ATP. Following treatment with matrine, we observed the decrease of cellular ATP level in a concentration-dependent manner as shown in **Figure 3B**.

Based on the molecular network regulated by matrine, we tried to infer the response of different cancer cell lines to endocytosis induced by matrine. We extracted the mRNA expression data of hepatocellular and colorectal cancer cell lines from the Cancer Cell Line Encyclopedia (Barretina et al., 2012). The result demonstrates that genes in endocytosis-associated network module of matrine are expressed more highly in DLD-1 cell line (**Figure 3C**). Then, we examined whether induction of cytoplasmic vacuolization by matrine is cell-specific. Therefore, we performed the same treatment experiments in various cancer cell lines. Strikingly, we observed more significant morphological changes with increasing concentrations of matrine in the DLD-1 than other cancer cell lines (**Figure 3D**).

### Matrine as a Novel Macropinocytosis Inducer in Cancer Cells

In fact, previous studies have demonstrated that matrine increases the cell volume and induces the formation of abundant cytoplasmic vacuoles in the SGC-7901 human gastric cancer cells and human hepatoma G2 cells (Zhang et al., 2010; Zhang J. et al., 2011), but how these vacuoles are formed is not fully understood. To further verify the effect of matrine on the regulation of membrane organization, we set out to determine the membrane changes in different cancer cells treated with matrine and also observed vesicle formation by endocytosis.

**FIGURE 4:**
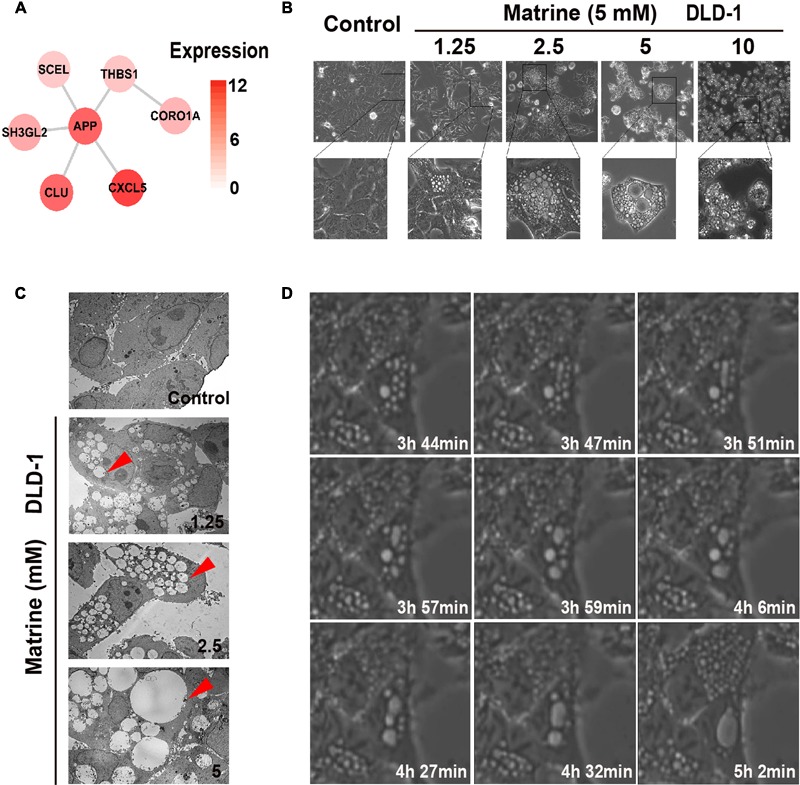
Matrine induces vesicle formation, endocytosis and macropinocytosis in a time-and concentration-dependent manner. **(A)** The endocytosis-associated network module of matrine with expression in DLD-1. **(B)** Phase-contrast microscopy of the matrine-treated DLD-1 cells for 12 h shows extensive accumulation of cytoplasmic vacuoles and cell detachment in a concentration-dependent manner. **(C)** TEM images of matrine-treated DLD-1 cells at different concentrations for 12 h. Red arrow points at cytoplasmic vacuoles. Bar, 10 μM. **(D)** Live imaging of DLD-1 cells (5 mM matrine). Images collected at times of major phenotypic changes.

The endocytosis-associated network module of matrine with expression in DLD-1 is shown in **Figure 4A**. The results suggest that high expression of these predicted targets of matrine in DLD-1 cells is associated with biological response to matrine. Therefore, observation of DLD-1 cells with standard phase contrast optics and a transmission electron microscope revealed that the biological processes initiated by the addition of matrine at increasing concentrations (1.25–10 mM) to DLD-1 cells within 24 h involve massive membrane ruffling and blebbing as well as cell rounding, followed by the formation of vacuoles that increase in number and size in a concentration-dependent manner (**Figures 4B,C**). 5 mM matrine was selected for further study because this concentration yielded the most significant number of vacuoles. Time-lapse phase-contrast microscopy was also used to capture the dynamic changes of cytoplasmic vacuolization over time within 6 h and found that nascent vesicles could fuse with each other to form progressively larger vacuoles within the cytoplasm (**Figure 4D**). The results confirm the induction of endocytic-like activity by matrine. Since macropinocytosis is defined as the formation of large endocytic vesicles of irregular sizes and shapes by cells that avidly incorporate extracellular fluid (Swanson, 2008; Kerr and Teasdale, 2009), we reasoned that this cellular process mediated by matrine could be macropinocytosis.

### Matrine-Induced Vacuoles Are Macropinosomes

**FIGURE 5:**
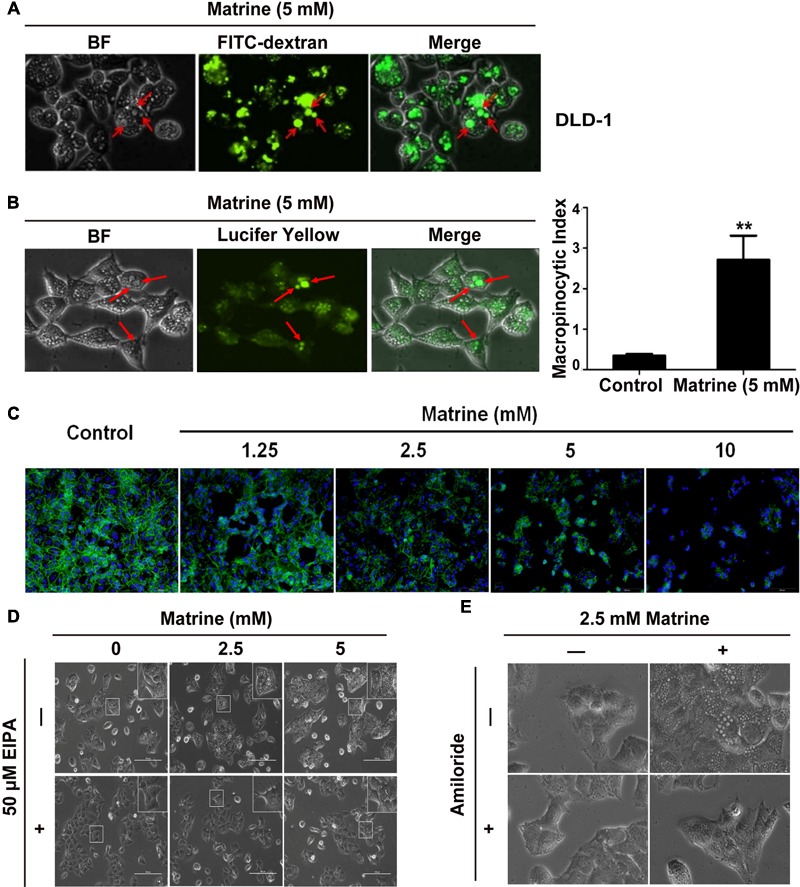
The vacuoles were derived from macropinosomes. **(A)** Vacuolated cells were preincu-bated with FITC-dextran (green). Arrowhead points to a FITC dextran-containing vacuole. **(B)** LY accumulation in DLD-1 cells (green). Arrowhead points to a LY-containing vacuole. Macropinocytosis index (MI) was ^∗∗^*P* < 0.01, compared with control. **(C)** DLD-1 cells were stimulated with 5 mM matrine. Nuclei were stained with DAPI (blue), and cells were stained for actin with rhodamine-phalloidin (red). **(D)** Effect of EIPA on matrine-induced macropinocytosis. **(E)** Effect of amiloride on matrine-induced macropinocytosis.

Macropinocytosis is known to be a form of actin-dependent endocytosis that leads to the internalization of fluid and membranes into large vesicles as macropinosomes. Next, we tested whether the large vesicles induced by matrine are consistent with the typical characteristics of macropinosome. As we know, rapid incorporation of extracellular-phase fluid tracers is a hallmark of macropinosomes (Swanson, 2008). Therefore, to confirm that the vacuoles were derived from macropinosomes, DLD-1 cells were subjected to short-term incubation with a bulk fluid-phase tracer, FITC-dextran, together with matrine. Our results showed that the uptake of FITC-dextrans by the vacuoles was almost equal during the 5 mM matrine treatment, indicating that the origins of many of these vacuoles could be macropinosomes (**Figure 5A**). To further confirm that the matrine-induced vacuoles observed by phase-contrast microscopy were indeed derived from macropinosomes, DLD-1 cells were incubated with the tracer LY during the first 5 h after the addition of matrine. As shown in **Figure 5B**, LY was also incorporated into most of the phase-lucent vacuoles. After fixing and staining with rhodamine-phalloidin, phalloidin counterstaining on DLD-1 cells shows that matrine disturbed the actin filament cytoskeleton of DLD-1 cells (**Figure 5C**). Macropinocytosis can be distinguished from some types of endocytosis by its susceptibility to EIPA and amiloride (inhibitors for Na+/H+ exchangers). We investigated the effects of EIPA and amiloride on macropinocytosis in DLD-1 cells. **Figures 5D,E** show that the cytoplasmic vacuolization during the 5 mM matrine treatment was markedly inhibited by 50 μM EIPA and 4 mM amiloride, respectively. We concluded that the origin of a substantial portion of the cytoplasmic vacuoles was macropinocytosis. These results demonstrated that macropinocytosis was involved in matrine-induced vacuolization and verified the efficiency of our computational method.

Macropinocytosis plays an important role in multifunctional biological processes, such as nutrient supply and non-apoptotic death of tumor cells (Commisso et al., 2013; Kitambi et al., 2014).

## Discussion

Experimental methods for target identification of natural products have had substantial success, yet many limitations still remain, including a high false-positive rate and bioactivity changes in labeled ligands (Sche et al., 1999; Terstappen et al., 2007). In recent years, many computational methods and algorithms have been developed to predict target profiles, such as phenotypic effect-based (Parsons et al., 2004; Campillos et al., 2008) and chemical structure-based approaches (Keiser et al., 2009). Iorio et al. (2010) developed a computation approach to predict drug effect similarities and modes of action using ‘consensus’ transcript signatures following compound treatment. However, their method is largely dependent on drugs with known transcriptional signatures, therefore limiting the application of this system in high-throughput compound screening. Cheng et al. (2012) proposed different methods to predict drug-target interactions and demonstrated that network-based methods achieved the best performance in benchmark tests (Cheng et al., 2012). Nevertheless, their method only considers proteins that are known targets. To identify global targets of natural products, we used a network-based target prediction approach, which is not limited to the structural information of target proteins and requires the chemical structure of natural products. This computational approach can meet the characteristics of mechanism of natural products. More broadly, we can also extend the application of our general network-based approach to identifying pharmacological functions of full-scale ingredients from one herb.

Several alkaloids, as the main active constituents of *Ku-Shen*, have been approved as drugs, including matrine, oxymatrine, and sophoridine. In fact, the biological activities of matrine are more relevant to other applications of *Ku-Shen* other than anti-tumor activities. It is reported to possess a variety of pharmacological activities, including antipyretic (Cho et al., 1986; Xiang and Jiang, 2013), antiepileptic (Xiang and Jiang, 2013), antinociceptive (Wang et al., 2013; Dun et al., 2014), anti-inflammatory (Zhang B. et al., 2011; Wu G. et al., 2017), myocardial preservation (Zhang et al., 2006), antifibrotic (Zhang et al., 2001), anti-tumor (Wu J. et al., 2017), anti-viral (Li et al., 2005) and anti-angiogenic (Li et al., 2010). Our predicted results demonstrated that matrine could target a molecular network related to multiple functions, which is consistent with the multi-target nature of traditional medicine. Therefore, matrine regulates ATP metabolism and the induction of vesicle formation and endocytosis by regulating the network target of matrine. The pharmacological functions of matrine based on network target and experimental verification reveal that the biological processes initiated by matrine involves massive membrane reorganization and cell rounding, followed by vacuole formation, which is a typical macropinocytosis process. Recently, macropinocytosis has been reported to be involved in the phagocytosis of apoptotic cells and promote the resolution of inflammation (Hoffmann et al., 2001; Ogden et al., 2001). Moreover, the small GTPase Rac1, which mediates the formation of the initial membrane ruffles and macropinosomes, is crucial for mammary alveolar epithelia to switch from secretion mode to phagocytic mode to rapidly remove dying neighbors and inhibit chronic inflammation (Akhtar et al., 2016). Therefore, a novel anti-inflammatory mechanism of matrine may be associated with the clearance of apoptotic cells by the macropinocytosis process. This finding can be used to better elucidate the “clearing hot” efficacy of *Ku-Shen* in TCM.

In order to further obtain the evidence of the mechanism of matrine, the observation that amiloride and EIPA can attenuate the macropinocytosis induced by matrine suggested that its mechanism may be correlated with the proton channel. The genes related to Vesicle formation and Endocytosis in the network target of matrine are APP, GRIA2, GRIA1, ATP5B, DLG4, MERTK, THBS1 and the genes in Endocytosis-associated module also include SCEL, THBS1, CORO1A, CXCL5, CLU, and SH3GL2. Therefore, we inferred that matrine-induced macropinocytosis occurs through ATP5B signaling or GRIA2 and GRIA1. These hypotheses need to be further validated prospectively.

In summary, we developed a network target strategy for comprehensive functions of matrine in a Chinese medical herb, Sophorae Flavescentis Radix (*Ku-Shen*), and provided experimental evidence to demonstrate the availability of a network-target-based approach. The network target regulated by matrine provides evidence for the diverse functions of matrine in *Ku-Shen* and shows that matrine can induce macropinocytosis in cancer cells and can decrease cellular ATP level. Our general network-based approach to fully elucidate comprehensive functions of matrine can be applied to many other natural products for the identification of their pharmacological functions.

## Author Contributions

SL and BZ designed and supervised the study. BZ, S-YW, MW, and YL performed the experiments, obtained and analyzed the data, and did the statistical analysis. XW and SL performed the computational analysis. All authors discussed the results and wrote the manuscript.

## Conflict of Interest Statement

The authors declare that the research was conducted in the absence of any commercial or financial relationships that could be construed as a potential conflict of interest. The reviewer TL and handling Editor declared their shared affiliation.

## References

[B1] Akhtar N. Li W. Mironov A. Streuli C. H. (2016). Rac1 controls both the secretory function of the mammary gland and its remodeling for successive gestations. *Dev. Cell* 38 522–535. 10.1016/j.devcel.2016.08.005 27623383 PMC5022528

[B2] Ashburner M. Ball C. A. Blake J. A. Botstein D. Butler H. Cherry J. M. (2000). Gene ontology: tool for the unification of biology. The gene ontology consortium. *Nat. Genet.* 25 25–29. 10.1038/75556 10802651 PMC3037419

[B3] Barretina J. Caponigro G. Stransky N. Venkatesan K. Margolin A. A. Kim S. (2012). The cancer cell line encyclopedia enables predictive modelling of anticancer drug sensitivity. *Nature* 483 603–607. 10.1038/nature11003 22460905 PMC3320027

[B4] Brehme M. Hantschel O. Colinge J. Kaupe I. Planyavsky M. Koecher T. (2009). Charting the molecular network of the drug target Bcr-Abl. *Proc. Natl. Acad. Sci. U.S.A.* 106 7414–7419. 10.1073/pnas.0900653106 19380743 PMC2670881

[B5] Burkard T. R. Rix U. Breitwieser F. P. Superti-Furga G. Colinge J. (2010). A computational approach to analyze the mechanism of action of the kinase inhibitor bafetinib. *PLOS Comput. Biol* 6:e1001001. 10.1371/journal.pcbi.1001001 21124949 PMC2987840

[B6] Campillos M. Kuhn M. Gavin A. Jensen L. J. Bork P. (2008). Drug target identification using side-effect similarity. *Science* 321 263–266. 10.1126/science.1158140 18621671

[B7] Cheng F. Liu C. Jiang J. Lu W. Li W. Liu G. (2012). Prediction of drug-target interactions and drug repositioning via network-based inference. *PLOS Comput. Biol.* 8:e1002503. 10.1371/journal.pcbi.1002503 22589709 PMC3349722

[B8] Cho C. H. Chuang C. Y. Chen C. F. (1986). Study of the antipyretic activity of matrine. A lupin alkaloid isolated from Sophora subprostrata. *Planta Med.* 5 343–345. 10.1055/s-2007-969179 3797498

[B9] Commisso C. Davidson S. M. Soydaner-Azeloglu R. G. Parker S. J. Kamphorst J. J. Hackett S. (2013). Macropinocytosis of protein is an amino acid supply route in Ras-transformed cells. *Nature* 497 633–637. 10.1038/nature12138 23665962 PMC3810415

[B10] Cordell G. A. (2014). Phytochemistry and traditional medicine-The revolution continues. *Phytochem. Lett.* 10 28–31. 10.1016/j.phytol.2014.06.002

[B11] Cui L. Cai Y. Cheng W. Liu G. Zhao J. Cao H. (2017). A novel, multi-target natural drug candidate, matrine, improves cognitive deficits in Alzheimer’s disease transgenic mice by inhibiting Aβ aggregation and blocking the RAGE/Abeta axis. *Mol. Neurobiol.* 54 1939–1952. 10.1007/s12035-016-9783-8 26899576

[B12] Drew A. K. Bensoussan A. Whyte I. M. Dawson A. H. Zhu X. Myers S. P. (2002). Chinese herbal medicine toxicology database: monograph on Radix Sophorae Flavescentis, “ku shen”. *J. Toxicol. Clin. Toxicol.* 40 173–176. 10.1081/CLT-120004406 12126189

[B13] Dun L. L. Li Y. X. Xu Y. Q. Zhou R. Ma L. Jin S. J. (2014). Antinociceptive effect of matrine on vincristine-induced neuropathic pain model in mice. *Neurol. Sci.* 35 815–821. 10.1007/s10072-013-1603-r6 24337989

[B14] Farha M. A. Brown E. D. (2016). Strategies for target identification of antimicrobial natural products. *Nat. Prod. Rep.* 33 668–680. 10.1039/c5np00127g 26806527

[B15] Hoffmann P. R. DeCathelineau A. M. Ogden C. A. Leverrier Y. Bratton D. L. Daleke D. L. (2001). Phosphatidylserine (PS) induces PS receptor-mediated macropinocytosis and promotes clearance of apoptotic cells. *J. Cell Biol.* 155 649–659. 10.1083/jcb.200108080 11706053 PMC2198875

[B16] Hopkins A. L. (2007). Network pharmacology. *Nat. Biotechnol.* 25 1110–1111. 10.1038/nchembio.118 17921993

[B17] Huang D. W. Sherman B. T. Lempicki R. A. (2009). Systematic and integrative analysis of large gene lists using DAVID bioinformatics resources. *Nat. Protoc.* 4 44–57. 10.1038/nprot.2008.211 19131956

[B18] Iorio F. Bosotti R. Scacheri E. Belcastro V. Mithbaokar P. Ferriero R. (2010). Discovery of drug mode of action and drug repositioning from transcriptional responses. *Proc. Natl. Acad. Sci. U.S.A.* 107 14621–14626. 10.1073/pnas.1000138107 20679242 PMC2930479

[B19] Kamei J. Xiao P. Ohsawa M. Kubo H. Higashiyama K. Takahashi H. (1997). Antinociceptive effects of (+)-matrine in mice. *Eur. J. Pharmacol.* 337 223–226. 10.1016/S0014-2999(97)01273-9 9430418

[B20] Keiser M. J. Setola V. Irwin J. J. Laggner C. Abbas A. I. Hufeisen S. J. (2009). Predicting new molecular targets for known drugs. *Nature* 462 175–181. 10.1038/nature08506 19881490 PMC2784146

[B21] Kerr M. C. Teasdale R. D. (2009). Defining macropinocytosis. *Traffic* 10 364–371. 10.1111/j.1600-0854.2009.00878.x 19192253

[B22] Kitambi S. S. Toledo E. M. Usoskin D. Wee S. Harisankar A. Svensson R. (2014). Vulnerability of glioblastoma cells to catastrophic vacuolization and death induced by a small molecule. *Cell* 157 313–328. 10.1016/j.cell.2014.02.021 24656405

[B23] Li C. Q. Zhu Y. T. Zhang F. X. Fu L. C. Li X. H. Cheng Y. (2005). Anti-HBV effect of liposome-encapsulated matrine *in vitro* and *in vivo*. *World J. Gastroenterol.* 11 426–428. 10.3748/wjg.v11.i3.426 15637760 PMC4205354

[B24] Li H. Tan G. Jiang X. Qiao H. Pan S. Jiang H. (2010). Therapeutic effects of matrine on primary and metastatic breast cancer. *Am. J. Chin. Med.* 38 1115–1130. 10.1142/S0192415X10008512 21061465

[B25] Li H. Zhao L. Zhang B. Jiang Y. Wang X. Guo Y. (2014). A network pharmacology approach to determine active compounds and action mechanisms of ge-gen-qin-lian decoction for treatment of type 2 diabetes. *Evid. Based Complement. Alternat. Med.* 2014:495840. 10.1155/2014/495840 24527048 PMC3914348

[B26] Li S. (2009). Network systems underlying traditional Chinese medicine syndrome and herb formula. *Curr. Bioinform.* 4 188–96. 10.2174/157489309789071129

[B27] Li S. (2011). Network target: a starting point for traditional Chinese medicine network pharmacology. *China J. Chin. Mater. Med.* 36 2017–2020. 10.4268/cjcmm20111502 22066431

[B28] Li S. (2015). Mapping ancient remedies: applying a network approach to traditional Chinese medicine. *Science* 350 S72–S74.

[B29] Li S. Zhang B. (2013). Traditional Chinese medicine network pharmacology: theory, methodology and application. *Chin. J. Nat Med.* 11 110–120. 10.1016/S1875-5364(13)60037-0 23787177

[B30] Li S. Zhang B. Zhang N. (2011). Network target for screening synergistic drug combinations with application to traditional Chinese medicine. *BMC Syst. Biol.* 5(Suppl. 1):S10. 10.1186/1752-0509-5-S1-S10 21689469 PMC3121110

[B31] Li S. Zhang Z. Wu L. Zhang X. Li Y. Wang Y. (2007). Understanding ZHENG in traditional Chinese medicine in the context of neuro-endocrine-immune network. *IET Syst. Biol.* 1 51–60. 10.1049/iet-syb:20060032 17370429

[B32] Liang C. Z. Zhang J. K. Shi Z. Liu B. Shen C. Q. Tao H. M. (2012). Matrine induces caspase-dependent apoptosis in human osteosarcoma cells in vitro and in vivo through the upregulation of Bax and Fas/FasL and downregulation of Bcl-2. *Cancer Chemother. Pharmacol.* 69 317–331. 10.1007/s00280-011-1699-4 21717192

[B33] Liang X. Li H. Li S. (2014). A novel network pharmacology approach to analyse traditional herbal formulae: the Liu-Wei-Di-Huang pill as a case study. *Mol. Biosyst.* 10 1014–1022. 10.1039/c3mb70507b 24492828

[B34] Liu J. Li J. (2012). Review on flavescens and its active ingredients in treatiment of insomnia. *Chin. J. Exp. Tradit. Med. Formulae* 18 284–288. 10.13422/j.cnki.syfjx.2012.11.077

[B35] Liu S. Q. Zhang M. L. Zhang H. J. Liu F. Z. Chu R. J. Zhang G. X. (2017). Matrine promotes oligodendrocyte development in CNS autoimmunity through the PI3K/Akt signaling pathway. *Life Sci.* 180 36–41. 10.1016/j.lfs.2017.05.010 28499934

[B36] Liu Y. Xu Y. Ji W. Li X. Sun B. Gao Q. (2014). Anti-tumor activities of matrine and oxymatrine: literature review. *Tumour. Biol.* 35 5111–5119. 10.1007/s13277-014-1680-z 24526416

[B37] Lu Z. Li M. Wang J. Wei D. Liu Q. Kong L. (2014). Developmental toxicity and neurotoxicity of two matrine-type alkaloids, matrine and sophocarpine, in zebrafish (*Danio rerio*) embryos/larvae. *Reprod. Toxicol.* 47 33–41. 10.1016/j.reprotox.2014.05.015 24911943

[B38] Ogden C. A. DeCathelineau A. Hoffmann P. R. Bratton D. Ghebrehiwet B. Fadok V. A. (2001). C1q and mannose binding lectin engagement of cell surface calreticulin and CD91 initiates macropinocytosis and uptake of apoptotic cells. *J. Exp. Med.* 194 781–795. 10.1084/jem.194.6.781 11560994 PMC2195958

[B39] Overmeyer J. H. Kaul A. Johnson E. E. Maltese W. A. (2008). Active ras triggers death in glioblastoma cells through hyperstimulation of macropinocytosis. *Mol. Cancer Res.* 6 965–977. 10.1158/1541-7786.MCR-07-2036 18567800 PMC2994605

[B40] Parsons B. Tive L. Huang S. (2004). Gabapentin: a pooled analysis of adverse events from three clinical trials in patients with postherpetic neuralgia. *Am. J. Geriatr. Pharmacother.* 2 157–162. 10.1016/j.amjopharm.2004.09.004 15561647

[B41] Qi Q. Li R. Li H. Cao Y. Bai M. Fan X. (2016). Identification of the anti-tumor activity and mechanisms of nuciferine through a network pharmacology approach. *Acta Pharmacol. Sin.* 37 963–972. 10.1038/aps.2016.53 27180984 PMC4933762

[B42] Rubio-Perez C. Tamborero D. Schroeder M. P. Antolin A. A. Deu-Pons J. Perez-Llamas C. (2015). In silico prescription of anticancer drugs to cohorts of 28 tumor types reveals targeting opportunities. *Cancer Cell* 27 382–396. 10.1016/j.ccell.2015.02.007 25759023

[B43] Sche P. P. McKenzie K. M. White J. D. Austin D. J. (1999). Display cloning: functional identification of natural product receptors using cDNA-phage display. *Chem. Biol.* 6 707–716. 10.1016/S1074-5521(00)80018-6 10508685

[B44] Swanson J. A. (2008). Shaping cups into phagosomes and macropinosomes. *Nat. Rev. Mol. Cell. Biol.* 9 639–649. 10.1038/nrm2447 18612320 PMC2851551

[B45] Terstappen G. C. Schlupen C. Raggiaschi R. Gaviraghi G. (2007). Target deconvolution strategies in drug discovery. *Nat. Rev. Drug Discov.* 6 891–903. 10.1038/nrd2410 17917669

[B46] Wang D. Cao Y. Zheng L. Lv D. Chen L. Xing X. (2017). Identification of Annexin A2 as a target protein for plant alkaloid matrine. *Chem. Commun.* 53 5020–5023. 10.1039/c7cc02227a 28428997

[B47] Wang H. Li Y. Dun L. Xu T. Hao Y. Liu H. (2013). Antinociceptive effects of matrine on neuropathic pain induced by chronic constriction injury. *Pharm. Biol.* 51 844–850. 10.3109/13880209.2013.767363 23627473

[B48] Wang X. Liu Y. (2012). Intervention effect of Matrine on catecholamine to left ventricular outflow tract pacemaker cells electrophysiological change. *Chin. J. Basic Med. Tradit. Chin. Med.* 18 1265–1267.

[B49] Wei Y. Tang H. Li X. (2013). Influence of matrine on sodium channel current of ventricle muscle cells in guinea pigs. *Chin. J. Exp. Tradit. Med. Formulae* 20 199–202.

[B50] Wu G. Zhou W. Zhao J. Pan X. Sun Y. Xu H. (2017). Matrine alleviates lipopolysaccharide-induced intestinal inflammation and oxidative stress via CCR7 signal. *Oncotarget* 8 11621–11628. 10.18632/oncotarget.14598 28086227 PMC5355291

[B51] Wu J. Hu G. Dong Y. Ma R. Yu Z. Jiang S. (2017). Matrine induces Akt/mTOR signalling inhibition-mediated autophagy and apoptosis in acute myeloid leukaemia cells. *J. Cell. Mol. Med.* 21 1171–1181. 10.1111/jcmm.13049 28026112 PMC5431164

[B52] Wu M. Lu P. Shi L. Li S. (2015). Traditional Chinese patent medicines for cancer treatment in China: a nationwide medical insurance data analysis. *Oncotarget* 6 38283–38295. 10.18632/oncotarget.5711 26513017 PMC4741999

[B53] Xiang J. Jiang Y. (2013). Antiepileptic potential of matrine via regulation the levels of gamma-aminobutyric acid and glutamic acid in the brain. *Int. J. Mol. Sci.* 14 23751–23761. 10.3390/ijms141223751 24317434 PMC3876075

[B54] Xu Z. (2011). Modernization: one step at a time. *Nature* 480 S90–S92. 10.1038/480S90a 22190089

[B55] Yin L. L. Zhu X. Z. (2005). The involvement of central cholinergic system in (+)-matrine-induced antinociception in mice. *Pharmacol. Biochem. Behav.* 80 419–425. 10.1016/j.pbb.2004.12.008 15740784

[B56] Zeng X. Wang H. Bai F. Zhou X. Li S. Ren L. (2015). Identification of matrine as a promising novel drug for hepatic steatosis and glucose intolerance with HSP72 as an upstream target. *Br. J. Pharmacol.* 172 4303–4318. 10.1111/bph.13209 26040411 PMC4556469

[B57] Zhang B. Liu Z. Y. Li Y. Y. Luo Y. Liu M. L. Dong H. Y. (2011). Antiinflammatory effects of matrine in LPS-induced acute lung injury in mice. *Eur. J. Pharm. Sci.* 44 573–579. 10.1016/j.ejps.2011.09.020 22019524

[B58] Zhang B. Lu C. Bai M. He X. Tan Y. Bian Y. (2015). Tetramethylpyrazine identified by a network pharmacology approach ameliorates methotrexate-induced oxidative organ injury. *J. Ethnopharmacol.* 175 638–647. 10.1016/j.jep.2015.09.034 26435225

[B59] Zhang J. Li Y. Chen X. Liu T. Chen Y. He W. (2011). Autophagy is involved in anticancer effects of matrine on SGC-7901 human gastric cancer cells. *Oncol. Rep.* 26 115–124. 10.3892/or.2011.1277 21519796

[B60] Zhang J. Su K. Shi W. Wang Y. Hu P. Wang Y. (2013). Matrine inhibits the adhesion and migration of BCG823 gastric cancer cells by affecting the structure and function of the vasodilator-stimulated phosphoprotein (VASP). *Acta Pharmacol. Sin.* 34 1084–1092. 10.1038/aps.2013.15 23685951 PMC4003018

[B61] Zhang J. P. Zhang M. Zhou J. P. Liu F. T. Zhou B. Xie W. F. (2001). Antifibrotic effects of matrine on *in vitro* and *in vivo* models of liver fibrosis in rats. *Acta. Pharmacol. Sin.* 22 183–186. 11741525

[B62] Zhang J. Q. Li Y. M. Liu T. He W. T. Chen Y. T. Chen X. H. (2010). Antitumor effect of matrine in human hepatoma G2 cells by inducing apoptosis and autophagy. *World J. Gastroenterol.* 16 4281–4290. 10.3748/wjg.v16.i34.4281 20818811 PMC2937108

[B63] Zhang X. Song J. (2014). Matrine suppresses glutamate excitotoxicity in experimental autoimmune encephalomyelitis. *J. Immunol* 560 92–97. 10.1016/j.neulet.2013.12.031 24368216

[B64] Zhang Y. J. Xiang M. X. San J. Cheng G. Wang S. S. (2006). Effect of matrine and carvedilol on collagen and MMPs activity of hypertrophy myocardium induced by pressure overload. *J. Zhejiang Univ. Sci. B* 7 245–250. 10.1631/jzus.2006.B0245 16502514 PMC1419068

[B65] Zhao S. Iyengar R. (2012). Systems pharmacology: network analysis to identify multiscale mechanisms of drug action. *Annu. Rev. Pharmacol. Toxicol.* 52 505–521. 10.1146/annurev-pharmtox-010611-134520 22235860 PMC3619403

[B66] Zhao S. Li S. (2010). Network-based relating pharmacological and genomic spaces for drug target identification. *PLOS ONE* 5:e11764. 10.1371/journal.pone.0011764 20668676 PMC2909904

[B67] Zhao X. Kan Q. Zhu L. Zhang G. X. (2011). Matrine suppresses production of IL-23/IL-17 and ameliorates experimental autoimmune encephalomyelitis. *Am. J. Chin. Med.* 39 933–941. 10.1142/S0192415X11009317 21905283

